# 3-Methyl­quinoxaline-2-carb­oxy­lic acid 4-oxide monohydrate

**DOI:** 10.1107/S160053681002266X

**Published:** 2010-06-26

**Authors:** Yubo Li, Wenfeng Zhou, Jinli Wang, Haixiang Gao, Zhiqiang Zhou

**Affiliations:** a Department of Applied Chemistry, China Agricultural University, Yuanmingyuan, West Road 2#, Haidian District, Beijing 100194, People’s Republic of China

## Abstract

In the crystal structure of the title compound, C_10_H_8_N_2_O_3_·H_2_O, mol­ecules are linked *via* inter­molecular O—H⋯O and O—H⋯N hydrogen bonds into a two-dimensional network.

## Related literature

For the synthesis of the starting material, see: Robertson & Kasublck (1973 ▶). For the synthesis of the title compound, see: Dirlam & McFarland (1977 ▶).
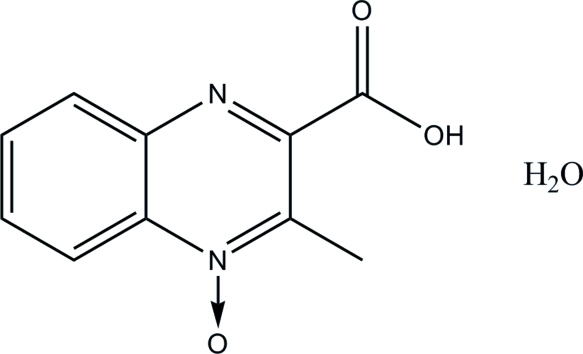


## Experimental

### 

#### Crystal data

C_10_H_8_N_2_O_3_·H_2_O
*M* *_r_* = 222.20
Monoclinic, 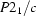

*a* = 6.0526 (13) Å
*b* = 18.068 (4) Å
*c* = 8.9195 (19) Å
β = 98.520 (15)°
*V* = 964.7 (4) Å^3^
*Z* = 4
Cu *K*α radiation
μ = 1.02 mm^−1^
*T* = 173 K
0.20 × 0.20 × 0.04 mm

#### Data collection

Rigaku R-AXIS RAPID IP area-detector diffractometer
Absorption correction: numerical (*ABSCOR*; Higashi, 1995 ▶) *T*_min_ = 0.822, *T*_max_ = 0.960
6140 measured reflections
1568 independent reflections
900 reflections with *I* > 2σ(*I*)
*R*_int_ = 0.077

#### Refinement

*R*[*F* ^2^ > 2σ(*F* ^2^)] = 0.098
*wR*(*F* ^2^) = 0.256
*S* = 1.10
1568 reflections
154 parameters
3 restraints
H atoms treated by a mixture of independent and constrained refinement
Δρ_max_ = 0.38 e Å^−3^
Δρ_min_ = −0.31 e Å^−3^

### 

Data collection: *RAPID-AUTO* (Rigaku, 2001 ▶); cell refinement: *RAPID-AUTO*; data reduction: *RAPID-AUTO*; program(s) used to solve structure: *SHELXS97* (Sheldrick, 2008 ▶); program(s) used to refine structure: *SHELXL97* (Sheldrick, 2008 ▶); molecular graphics: *SHELXTL* (Sheldrick, 2008 ▶); software used to prepare material for publication: *SHELXTL*.

## Supplementary Material

Crystal structure: contains datablocks I, global. DOI: 10.1107/S160053681002266X/lh5056sup1.cif

Structure factors: contains datablocks I. DOI: 10.1107/S160053681002266X/lh5056Isup2.hkl

Additional supplementary materials:  crystallographic information; 3D view; checkCIF report

## Figures and Tables

**Table 1:** Hydrogen-bond geometry (Å, °)

*D*—H⋯*A*	*D*—H	H⋯*A*	*D*⋯*A*	*D*—H⋯*A*
O1*W*—H1*WB*⋯O1^i^	0.85 (4)	1.97 (2)	2.794 (5)	164 (5)
O1*W*—H1*WA*⋯N2^ii^	0.86 (4)	2.14 (2)	2.968 (5)	163 (5)
O3—H3⋯O1*W*	0.84	1.76	2.574 (5)	162

Symmetry codes: (i) 
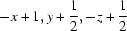
; (ii) 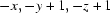.

**Fig. 1.**
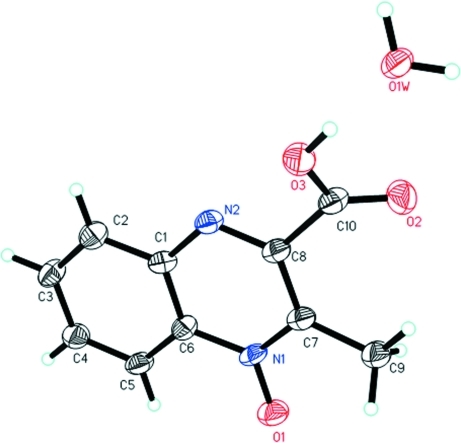
The molecular structure of the title compound, showing the labelling scheme. Displacement ellipsoids are drawn at the 30% probability level for all non-H atoms.

**Fig. 2.**
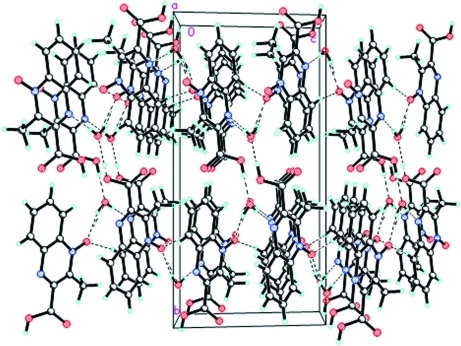
Part of the crystal structure of the title compound viewed along the a axis. Hydrogen bonds are shown as dashed lines.

## supplementary crystallographic information

### Comment

The molecular structure of the title compound is shown in Fig. 1. In the crystal structure, molecules are linked via intermolecular O-H···O hydrogen bonds into a two-dimensional network.

### Experimental

Following the procedure of Dirlam & McFarland (1977) ethyl-3-methyl-2-quinoxalinecarboxylate-1,4-dioxide (2.0 g, 8 mmol) (Robertson & Kasublck, 1973) was dissolved in 1-propanol (20 ml), trimethyl phosphate (2.0 g, 16 mmol) was added dropwise to the solution. The reaction mixture was heated under reflux for 2.5 h, and evaporated to dryness. The residue was recrystallized from ether-hexane (1:1) to yeild 1.6 g (80%) ethyl 2-methyl-3-quinoxalinecarboxylate-1-oxide. Ethyl 2-methyl-3-quinoxalinecarboxylate-1-oxide (5 g, 22 mmol) was suspended in aqueous 0.5*M* sodium hydroxide solution (50 mL), and stirred for 2 h. Then used concentrated hydrochloric acid to adjust the PH=2. The white solid was collected and recrystallized from water to give 4.0 g (80%) of the the title compound.

### Refinement

All H atoms (except for those bonded to the solvent water) were placed in calculated positions C-H = 0.95-0.98Å; O-H = 0.86Å and refined in a riding-model approximation with U_iso_(H) = 1.2U_eq_(C) or 1.5U_eq_(C_methyl_,O). The H atoms of the solvent water molecule were located in a difference Fourier and there positions were refined with restraints and with U_iso_(H) = 1.5U_eq_(O).

The crystals of the title compound were of low quality and the data used has resulted in a crystal structure which has lower than normal precision. The precision of the data however is adequate to describe the nature of the hydrogen bonding.

### Crystal data

**Table:**

C_10_H_8_N_2_O_3_·H_2_O	*F*(000) = 464
*M**_r_* = 222.20	*D*_x_ = 1.530 Mg m^−^^3^
Monoclinic, *P*2_1_/*c*	Cu *K*α radiation, λ = 1.54186 Å
Hall symbol: -P 2ybc	Cell parameters from 426 reflections
*a* = 6.0526 (13) Å	θ = 3.1–68.1°
*b* = 18.068 (4) Å	µ = 1.02 mm^−^^1^
*c* = 8.9195 (19) Å	*T* = 173 K
β = 98.520 (15)°	Plate, yellow
*V* = 964.7 (4) Å^3^	0.20 × 0.20 × 0.04 mm
*Z* = 4	

### Data collection

**Table:**

Rigaku R-AXIS RAPID IP area-detector diffractometer	1568 independent reflections
Radiation source: fine-focus sealed tube	900 reflections with *I* > 2σ(*I*)
graphite	*R*_int_ = 0.077
ω scans at fixed χ = 45°	θ_max_ = 63.7°, θ_min_ = 4.9°
Absorption correction: numerical (*ABSCOR*; Higashi, 1995)	*h* = −7→7
*T*_min_ = 0.822, *T*_max_ = 0.960	*k* = −20→20
6140 measured reflections	*l* = −10→9

### Refinement

**Table:**

Refinement on *F*^2^	Secondary atom site location: difference Fourier map
Least-squares matrix: full	Hydrogen site location: inferred from neighbouring sites
*R*[*F*^2^ > 2σ(*F*^2^)] = 0.098	H atoms treated by a mixture of independent and constrained refinement
*wR*(*F*^2^) = 0.256	*w* = 1/[σ^2^(*F*_o_^2^) + (0.1186*P*)^2^ + 0.0706*P*] where *P* = (*F*_o_^2^ + 2*F*_c_^2^)/3
*S* = 1.10	(Δ/σ)_max_ < 0.001
1568 reflections	Δρ_max_ = 0.38 e Å^−^^3^
154 parameters	Δρ_min_ = −0.31 e Å^−^^3^
3 restraints	Extinction correction: *SHELXL97* (Sheldrick, 2008), Fc^*^=kFc[1+0.001xFc^2^λ^3^/sin(2θ)]^-1/4^
Primary atom site location: structure-invariant direct methods	Extinction coefficient: 0.009 (2)

### Special details

**Table:**

Geometry. All e.s.d.'s (except the e.s.d. in the dihedral angle between two l.s. planes) are estimated using the full covariance matrix. The cell e.s.d.'s are taken into account individually in the estimation of e.s.d.'s in distances, angles and torsion angles; correlations between e.s.d.'s in cell parameters are only used when they are defined by crystal symmetry. An approximate (isotropic) treatment of cell e.s.d.'s is used for estimating e.s.d.'s involving l.s. planes.
Refinement. Refinement of *F*^2^ against ALL reflections. The weighted *R*-factor *wR* and goodness of fit *S* are based on *F*^2^, conventional *R*-factors *R* are based on *F*, with *F* set to zero for negative *F*^2^. The threshold expression of *F*^2^ > σ(*F*^2^) is used only for calculating *R*-factors(gt) *etc*. and is not relevant to the choice of reflections for refinement. *R*-factors based on *F*^2^ are statistically about twice as large as those based on *F*, and *R*- factors based on ALL data will be even larger.

### Fractional atomic coordinates and isotropic or equivalent isotropic displacement parameters (Å^2^)

**Table:**

	*x*	*y*	*z*	*U*_iso_*/*U*_eq_	
O1W	0.2035 (6)	0.6094 (2)	0.4980 (5)	0.0458 (12)	
O1	0.6172 (6)	0.2378 (2)	0.1107 (4)	0.0546 (12)	
O2	0.3088 (6)	0.4949 (2)	0.2089 (5)	0.0614 (13)	
O3	0.2260 (7)	0.4704 (2)	0.4389 (4)	0.0532 (12)	
H3	0.2018	0.5162	0.4401	0.080*	
N1	0.4745 (7)	0.2663 (3)	0.1891 (5)	0.0393 (12)	
N2	0.1661 (7)	0.3286 (2)	0.3563 (5)	0.0389 (12)	
C1	0.1772 (8)	0.2529 (3)	0.3378 (6)	0.0403 (14)	
C2	0.0313 (9)	0.2070 (3)	0.4034 (6)	0.0461 (15)	
H2	−0.0732	0.2280	0.4609	0.055*	
C3	0.0398 (9)	0.1315 (3)	0.3845 (6)	0.0474 (16)	
H3A	−0.0604	0.1004	0.4277	0.057*	
C4	0.1961 (9)	0.1006 (3)	0.3014 (6)	0.0490 (16)	
H4	0.2012	0.0483	0.2900	0.059*	
C5	0.3405 (9)	0.1436 (3)	0.2369 (6)	0.0451 (16)	
H5	0.4457	0.1219	0.1810	0.054*	
C6	0.3311 (8)	0.2207 (3)	0.2547 (6)	0.0387 (14)	
C7	0.4655 (9)	0.3408 (3)	0.2072 (6)	0.0402 (15)	
C8	0.3058 (9)	0.3687 (3)	0.2920 (6)	0.0383 (14)	
C9	0.6295 (9)	0.3835 (3)	0.1352 (6)	0.0482 (16)	
H9B	0.7788	0.3625	0.1647	0.072*	
H9C	0.6287	0.4352	0.1684	0.072*	
H9A	0.5896	0.3812	0.0247	0.072*	
C10	0.2818 (9)	0.4516 (3)	0.3070 (7)	0.0434 (15)	
H1WB	0.261 (8)	0.643 (2)	0.450 (6)	0.065*	
H1WA	0.080 (5)	0.626 (3)	0.522 (6)	0.065*	

### Atomic displacement parameters (Å^2^)

**Table:**

	*U*^11^	*U*^22^	*U*^33^	*U*^12^	*U*^13^	*U*^23^
O1W	0.041 (2)	0.044 (3)	0.056 (3)	−0.003 (2)	0.0210 (19)	−0.003 (2)
O1	0.049 (2)	0.054 (3)	0.065 (3)	0.007 (2)	0.021 (2)	−0.005 (2)
O2	0.081 (3)	0.050 (3)	0.059 (3)	0.009 (2)	0.030 (2)	0.008 (2)
O3	0.065 (3)	0.043 (3)	0.056 (3)	0.004 (2)	0.021 (2)	−0.002 (2)
N1	0.032 (2)	0.052 (3)	0.037 (3)	0.005 (2)	0.016 (2)	−0.004 (2)
N2	0.034 (2)	0.040 (3)	0.045 (3)	0.001 (2)	0.013 (2)	−0.006 (2)
C1	0.031 (3)	0.043 (4)	0.048 (3)	0.004 (3)	0.010 (3)	−0.004 (3)
C2	0.044 (3)	0.051 (4)	0.045 (3)	−0.005 (3)	0.010 (3)	−0.007 (3)
C3	0.045 (4)	0.046 (4)	0.056 (4)	−0.011 (3)	0.021 (3)	−0.008 (3)
C4	0.050 (4)	0.040 (4)	0.060 (4)	0.004 (3)	0.018 (3)	−0.003 (3)
C5	0.037 (3)	0.048 (4)	0.053 (4)	0.006 (3)	0.018 (3)	−0.004 (3)
C6	0.037 (3)	0.044 (4)	0.036 (3)	0.000 (3)	0.012 (3)	−0.002 (3)
C7	0.033 (3)	0.049 (4)	0.040 (3)	0.004 (3)	0.008 (2)	−0.003 (3)
C8	0.034 (3)	0.046 (4)	0.036 (3)	0.004 (3)	0.008 (2)	−0.002 (3)
C9	0.045 (4)	0.047 (4)	0.056 (4)	0.003 (3)	0.016 (3)	−0.002 (3)
C10	0.038 (3)	0.047 (4)	0.048 (4)	0.000 (3)	0.015 (3)	−0.006 (3)

### Geometric parameters (Å, °)

**Table:**

O1W—H1WB	0.85 (4)	C2—H2	0.9500
O1W—H1WA	0.86 (4)	C3—C4	1.401 (7)
O1—N1	1.296 (5)	C3—H3A	0.9500
O2—C10	1.202 (6)	C4—C5	1.359 (7)
O3—C10	1.316 (6)	C4—H4	0.9500
O3—H3	0.8400	C5—C6	1.404 (7)
N1—C7	1.357 (7)	C5—H5	0.9500
N1—C6	1.388 (6)	C7—C8	1.406 (7)
N2—C8	1.309 (6)	C7—C9	1.477 (7)
N2—C1	1.380 (6)	C8—C10	1.514 (8)
C1—C6	1.401 (6)	C9—H9B	0.9800
C1—C2	1.401 (7)	C9—H9C	0.9800
C2—C3	1.377 (6)	C9—H9A	0.9800
			
H1WB—O1W—H1WA	108 (4)	C6—C5—H5	120.6
C10—O3—H3	109.5	N1—C6—C1	118.8 (5)
O1—N1—C7	120.0 (5)	N1—C6—C5	120.3 (5)
O1—N1—C6	120.0 (5)	C1—C6—C5	120.9 (5)
C7—N1—C6	120.1 (4)	N1—C7—C8	117.5 (5)
C8—N2—C1	116.8 (4)	N1—C7—C9	115.2 (5)
N2—C1—C6	121.6 (5)	C8—C7—C9	127.3 (5)
N2—C1—C2	119.4 (5)	N2—C8—C7	125.2 (5)
C6—C1—C2	119.0 (5)	N2—C8—C10	115.6 (5)
C3—C2—C1	119.9 (5)	C7—C8—C10	119.0 (5)
C3—C2—H2	120.1	C7—C9—H9B	109.5
C1—C2—H2	120.1	C7—C9—H9C	109.5
C2—C3—C4	120.0 (5)	H9B—C9—H9C	109.5
C2—C3—H3A	120.0	C7—C9—H9A	109.5
C4—C3—H3A	120.0	H9B—C9—H9A	109.5
C5—C4—C3	121.5 (5)	H9C—C9—H9A	109.5
C5—C4—H4	119.3	O2—C10—O3	124.3 (6)
C3—C4—H4	119.3	O2—C10—C8	123.7 (5)
C4—C5—C6	118.7 (5)	O3—C10—C8	112.0 (5)
C4—C5—H5	120.6		
			
C8—N2—C1—C6	0.2 (7)	C4—C5—C6—C1	0.4 (8)
C8—N2—C1—C2	−179.8 (5)	O1—N1—C7—C8	179.4 (4)
N2—C1—C2—C3	179.4 (4)	C6—N1—C7—C8	−0.7 (7)
C6—C1—C2—C3	−0.6 (8)	O1—N1—C7—C9	−1.3 (7)
C1—C2—C3—C4	0.9 (8)	C6—N1—C7—C9	178.7 (4)
C2—C3—C4—C5	−0.6 (8)	C1—N2—C8—C7	−0.4 (8)
C3—C4—C5—C6	0.0 (8)	C1—N2—C8—C10	176.9 (4)
O1—N1—C6—C1	−179.5 (4)	N1—C7—C8—N2	0.6 (8)
C7—N1—C6—C1	0.6 (7)	C9—C7—C8—N2	−178.7 (5)
O1—N1—C6—C5	0.2 (7)	N1—C7—C8—C10	−176.5 (4)
C7—N1—C6—C5	−179.7 (4)	C9—C7—C8—C10	4.2 (8)
N2—C1—C6—N1	−0.3 (7)	N2—C8—C10—O2	−143.8 (5)
C2—C1—C6—N1	179.6 (5)	C7—C8—C10—O2	33.6 (8)
N2—C1—C6—C5	180.0 (5)	N2—C8—C10—O3	35.6 (6)
C2—C1—C6—C5	−0.1 (8)	C7—C8—C10—O3	−147.0 (5)
C4—C5—C6—N1	−179.4 (5)		

### Hydrogen-bond geometry (Å, °)

**Table:**

*D*—H···*A*	*D*—H	H···*A*	*D*···*A*	*D*—H···*A*
O1W—H1WB···O1^i^	0.85 (4)	1.97 (2)	2.794 (5)	164 (5)
O1W—H1WA···N2^ii^	0.86 (4)	2.14 (2)	2.968 (5)	163 (5)
O3—H3···O1W	0.84	1.76	2.574 (5)	162

Symmetry codes: (i) −*x*+1, *y*+1/2, −*z*+1/2; (ii) −*x*, −*y*+1, −*z*+1.
